# Attending to auditory memory changes with age

**DOI:** 10.18632/aging.101505

**Published:** 2018-07-25

**Authors:** Claude Alain, Linda Garami, Kristina C. Backer

**Affiliations:** 1 Rotman Research Institute, Baycrest Centre, Toronto, Ontario, Canada; 2 Department of Psychology University of Toronto, Ontario, Canada; 3 Institute of Medical Sciences, University of Toronto, Ontario, Canada; 4 School of Music, University of Toronto, Ontario, Canada; 5 Cognitive and Information Sciences, University of California, Merced, CA 95340, USA

**Keywords:** attention, working memory, aging

Speech comprehension in noisy environments, such as a busy restaurant, becomes increasingly challenging as we get older. Consequently, many older adults shy away from these complex auditory situations, which can lead to social isolation [1]. To date, research has identified multiple levels–from sensory to higher cognition–at which aging may negatively impact speech-in-noise comprehension. In the sensory (afferent) pathways, age-related changes in the peripheral auditory system (e.g., cochlea) can lead to hearing loss, hindering initial sound registration. Furthermore, a loss of information as the auditory signal travels from the ear to the brain could also interfere with the encoding and retrieval of sound object representations. A reduction in neural specificity may also decrease the distinctiveness between neural representations upstream and distort the hierarchy of speech representations. This in turn may impair the acoustic-phonetic mapping that is necessary for understanding speech. Inaccurate mapping may be a result of, or may interact with, efferent processes, such as age-related declines in the ability to regulate top-down attention to speech representations. In adverse listening conditions, selective attention may “refresh” (bring to the foreground) [2,3] or allow for the rehearsal [4] of the task-relevant representation in auditory short-term memory (ASTM), thereby easing speech comprehension. Previous research, using retro-cues (i.e., attentional cues presented after the auditory stimuli and during a silent retention interval, thereby directing attention to a particular auditory memory trace), have demonstrated that attention can be re-focused on internal memory representations and that past experiences encoded in long-term memory play a large role in shaping auditory perception [5,6]. However, research assessing the effects of age and hearing loss on the interaction between attention and memory is currently lacking. The research to date has focused primarily on perceptual and cognitive processes that operate on “incoming” external stimuli, and speech-in-noise assessments seldom take attention and memory processes into account.

To demonstrate the importance of the interaction between attention and memory during auditory scene analysis and to assess age differences in this interaction, Alain et al. [7] used a delayed match-to-sample task. Participants were presented with two auditory scenes that comprised three different everyday sounds

presented simultaneously from three different locations. During the retention interval, participants were presented with a retro-cue indicating which item, from a three-item memory array, should be held in ASTM. Younger adults took advantage of this information, and they oriented their attention toward the cued item in ASTM. In young adults, retrospectively attending to an item in ASTM was associated with enhanced event-related potential amplitude and decreased alpha and low beta power. These neural modulations were attenuated in older adults. These results suggest that older adults may have difficulty in orienting attention to a specific item in ASTM. For instance, older adults may not have as much control over their attentional spotlight as younger adults. One possibility is that older adults are more reluctant than young adults to “leave out” information and by default, use a more effortful strategy of maintaining all, even task-irrelevant, items in ASTM. In other words, older adults may have held all three items in ASTM, despite being retro-cued to the one task-relevant item.

Further research on the dynamic interactions between attention and memory is important for understanding how sound objects are represented, maintained, and accessed in the brain.

In the Alain et al. study, the task was fairly challenging because three environmental sounds from three different locations were presented simultaneously. Hence, it remains unclear whether older adults would benefit from a retro-cue to orient their attention to short-term memory if fewer items were presented. Fewer auditory objects might ease the segregation and representation of the incoming auditory signal in ASTM. A better understanding of these mechanisms could help mitigate auditory-related communication problems encountered by aging individuals, and the social isolation that is often associated with them. The knowledge gained from studies on attention to auditory memory should foster the development of interventions using neurostimulation and/or behavioral techniques that could delay or prevent these age-related changes. In summary, Alain et al.’s findings underscore the importance of attention to memory in understanding age-related changes in processing real-world auditory environments and pave the way for new avenues of inquiry into aging and auditory cognition.
